# 4Pi-RESOLFT nanoscopy

**DOI:** 10.1038/ncomms10504

**Published:** 2016-02-01

**Authors:** Ulrike Böhm, Stefan W. Hell, Roman Schmidt

**Affiliations:** 1 Department of NanoBiophotonics, Max Planck Institute for Biophysical Chemistry, Am Fassberg 11, Göttingen 37077, Germany

## Abstract

By enlarging the aperture along the optic axis, the coherent utilization of opposing objective lenses (4Pi arrangement) has the potential to offer the sharpest and most light-efficient point-spread-functions in three-dimensional (3D) far-field fluorescence nanoscopy. However, to obtain unambiguous images, the signal has to be discriminated against contributions from lobes above and below the focal plane, which has tentatively limited 4Pi arrangements to imaging samples with controllable optical conditions. Here we apply the 4Pi scheme to RESOLFT nanoscopy using two-photon absorption for the on-switching of fluorescent proteins. We show that in this combination, the lobes are so low that low-light level, 3D nanoscale imaging of living cells becomes possible. Our method thus offers robust access to densely packed, axially extended cellular regions that have been notoriously difficult to super-resolve. Our approach also entails a fluorescence read-out scheme that translates molecular sensitivity to local off-switching rates into improved signal-to-noise ratio and resolution.

## Abstract (web summary)

The ability to discriminate objects along the optic axis is a benchmark for three-dimensional imaging techniques. Here, the authors combine metastable-state switching and opposing objective lenses to suppress out-of-focus background and record three-dimensional nanoscale images of living cells.

## Graphical abstract

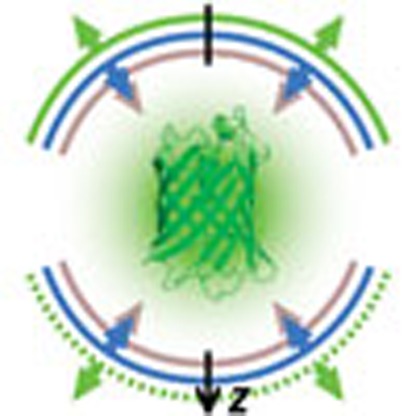

The three to seven fold improved axial resolution provided by 4Pi microscopy^1^,^2^,^3^ in the 1990s marked a first step in the quest for radically improving the resolution in far-field fluorescence microscopy. Yet the resolution provided by 4Pi microscopy remained diffraction-limited, because by jointly using two opposing lenses for focusing the excitation and/or the fluorescence light, this method just optimized the focusing conditions for feature separation. Modern far-field fluorescence nanoscopy^4^, or superresolution microscopy, such as the methods called stimulated emission depletion (STED), reversible fluorescent saturable optical transition (RESOLFT) and later also photoactivated localization microscopy (PALM)/stochastical optical reconstruction microscopy (STORM) fundamentally departed from such early superresolution concepts by discerning features through a molecular state transition. The use of a state transition for feature separation, typically a transition between a fluorescent (ON) and a non-fluorescent (OFF) state, opened the road to lens-based fluorescence microscopy with resolution that is conceptually not limited by diffraction.

Yet diffraction plays a role in these ‘diffraction-unlimited' techniques because the resolution of these ‘nanoscopy' methods still benefits strongly from focusing the light as sharply as possible. While in STED and RESOLFT, it is the focusing of the illumination light in sample space that matters, in PALM/STORM it is the focusing of the emitted light at the detector. Therefore, the optimization of focusing remains very timely. 4Pi arrangements can also facilitate the doubling of the detected fluorescence without compromising the resolution in the focal plane (*x*,*y*), and offer significantly sharper axial (*z*) intensity gradients than single lenses for both the illumination and the detected light. Consequently, the combination of 4Pi with STED, RESOLFT and PALM/STORM approaches currently offers the most powerful optical setting for three-dimensional (3D) fluorescence nanoscopy^5^,^6^,^7^.

Yet 4Pi-type super-resolution arrangements are scarcely reported for STED and PALM and entirely unexplored for RESOLFT, a STED-derivative that typically uses reversibly switchable fluorescent proteins (RSFP) for providing the mandatory ON and OFF states. RSFP-based RESOLFT is particularly attractive because it operates with low light levels, making it gentle to living cells^8^.

The difficulties of realizing a 4Pi setup are commonly attributed to the counter-alignment of the two high numerical aperture (NA) lenses. In practice, however, the alignment can be controlled and stabilized over many hours. Instead, a far more general problem that is inherent to all fluorescent imaging modalities comes to the fore. The fluorescence signal (that emanates from each sub-diffraction pixel volume under investigation) needs to be discriminated against ‘background' signal from outside of this volume. This ‘background' largely stems from optical aberrations that preclude precise spatial control of the illumination or fluorescence beam positions and, in case of STED, RESOLFT or PALM/STORM, from imperfections of the ON/OFF-state transfer (switching) process. Discrimination against this ‘background' signal is most challenging along the optical (*z*) axis, especially when the probed volume is of sub-diffraction dimensions. Lack of sufficient discrimination along the *z*-axis (optical sectioning) manifests itself as artifacts in the image, particularly as ‘ghost features' above and below the real features.

When describing the imaging process in the spatial frequency domain, the appearance of axial lobes corresponds to local depressions in the amplitude of the optical transfer function, that is, the modulation transfer function (MTF) of the microscope. Structural information of the sample can only be retrieved in those spatial frequency bands where the MTF is strong enough to convey a signal that sufficiently exceeds the local noise level. In a 4Pi microscope, MTF depressions are typically restricted to sharp local minima at the so-called critical frequencies^9^. As their amplitude strongly depends on the aperture angle *α* of the objective lenses used^9^, combinations of 4Pi with diffraction-unlimited super-resolution/nanoscopy methods such as isoSTED^5^,^10^,^11^ and iPALM^6^,^12^, have unfortunately been limited to imaging fixed samples that are more easily accessible with high angle lenses (*α*≥74°, as for oil immersion lenses with NA≥1.46). Furthermore, the imaged objects were rather thin and labelled very sparsely, as both properties alleviate the requirements on optical sectioning, that is, on suppressing (‘background') signal from above and below the focal plane. Fortunately, in a coordinate-targeted nanoscopy method such as RESOLFT, the signal received from the targeted nanosized pixel volume scales with the average number of molecules located within this volume, allowing for tailoring of the pixel volume^11^, and hence the resolution and the signal, to the actual imaging conditions to render the ‘background' (mathematically) treatable.

Here we report the realization of 4Pi-RESOLFT nanoscopy, that is, of a conceptually diffraction-unlimited resolving method which, by virtue of 4Pi microscopy, provides spatially uniform 3D resolution for imaging (living) cells at the nanometre scale, offers strong optical sectioning due to multiple background suppressing mechanisms and operates at low light levels in 3D.

## Results

### On-switching order and optical sectioning

The effective point-spread-function (PSF) *h*_ef_(**r**) of a coordinate-targeted superresolution imaging modality ultimately quantifies the 3D-coordinate range where the fluorescent molecules are allowed to yield measurable signal. If the fluorophores from a certain range are imaged onto a (confocal point) detector, *h*_ef_(**r**) is given by the normalized distribution *S*^ON^(**r**) showing where a molecule is allowed to be in the ON-state at the time of read-out, multiplied by a normalized function *h*^det^(**r**) that describes the detection probability:


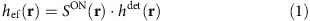


*S*_ON_(**r**) is proportional to a product of normalized terms *h*^on^ and 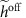 that describe the generation of the ON-state by the use of on- and off-switching processes, respectively. *h*^on^ describes the spatial probability to assume the ON-state in the absence of off-switching light. It can typically be written as a product of terms 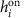 that each express the relative probability for absorption of a single photon that drives a transition to a (virtual) state, and therefore scales with the intensity of the light patterns used (for example, 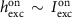 for single-photon excitation with intensity 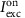; in case of two-photon excitation: 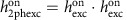). 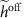 describes the effect of the off-switching light on a potential ON-state distribution; 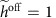 where molecules are always allowed to assume the ON-state, and 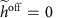 where they are forced to stay in an OFF-state. Due to the forced assumption of an OFF-state by ‘saturating' off-switching, 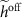 is usually much sharper than the off-switching light intensity patterns.

We formally define *h*^det^ as the first on-switching term 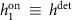 (because of its similar effect on *h*_ef_), drop any 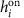 that does not significantly sharpen *h*_ef_ (for example, widefield-detection or sample-wide switching) and obtain:


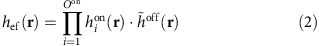


Here the number of on-factors *O*^on^ denotes the on-(switching) order of *h*_ef_, for example, *O*^on^=2 (excitation by single-photon absorption and confocal detection) for a typical confocal (STED) microscope. Optical sectioning can generally be improved by engineering 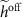 such that molecules in out-of-focus areas are switched off more effectively (Supplementary Fig. 1), or by requiring the absorption of multiple photons for the occupation of the ON-state, which increases *O*^on^. The latter can be realized directly through standard two-photon absorption^13^,^14^,^15^,^16^,^17^, or by requiring the sequential occupation of multiple real states to reach the ON-state^18^. Such sequential state occupation is easily realized using the switching steps offered by RSFP (that are central to the RSFP-based RESOLFT concept^4^,^19^).

### The 4Pi-RESOLFT modality

**Figure 1:**
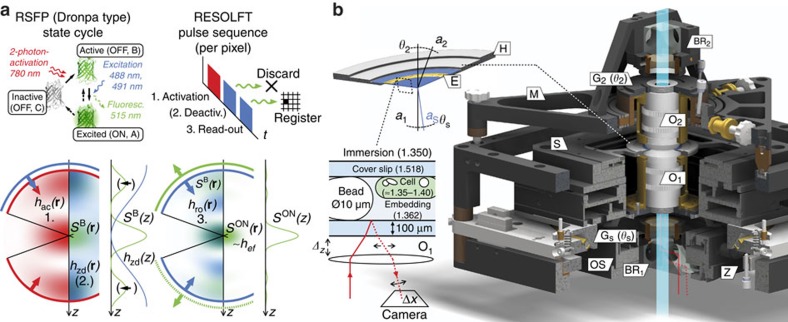
4Pi-RESOLFT principle and sample optics. (**a**) Coherent double-lens illumination cycles RSFP markers between dark (OFF) and bright (ON) states to generate spatial ON/OFF-contrast. For each pixel, an activation light pulse (focal pattern *h*_ac_) induces two-photon activation of RSFP (state transition C->B) in a pattern *S*^B^(**r**) with axial side-maxima (lobes) that are optionally suppressed by a subsequently applied deactivation pulse (*h*_zd_, B<->A->C). Fluorescence generated by the ON-state A is detected during read-out (B<->A->C) by a pattern *h*_ro_. Its mutual overlap with *S*^B^(**r**) is constrained to the focal centre, resulting in an effective PSF *h*_ef_∼*S*^ON^ that exhibits ≈100 nm axial FWHM and exceptionally low side-maxima. Profiles show on-axis values. (**b**) The upright 4Pi unit of the microscope. Cells are mounted on a ring-shaped sample holder (H), between two cover glasses fixed at 10 μm distance by spacer beads and epoxy resin (E). The set of refractive indices (in brackets) of the immersion and embedding medium, cover slip thickness and correction collar settings of the objective lenses (O_1_, O_2_) diminishes aberrations from the sample. The sample stage (S) is mounted on a vertically movable (Z) goniometer (G_S_), accepts the sample holder (H) and provides five degrees-of-freedom for coarse *xyz*-positioning and *z*-scanning of the sample, as well as tip-/tilt-alignment (*θ*_S_) of the cover slip normal (*a*_S_) to the optic axis of O_1_ (*a*_1_). O_1_ itself is mounted on a *xyz*-piezo stage (OS) that provides online fine control over the displacement of both foci. A triangular mount (M) allows for tip/tilt-(*θ*_2_) and coarse *xyz*-alignment of O_2_ (axis *a*_2_) with respect to O_1_, and can conveniently be detached to change the sample. Two polarizing beam splitter/quarter-wave retarder pairs (BR_1,2_) clean up and tune the polarization of the incident beam pairs to opposing circular states. One beam splitter furthermore serves as a port for an alignment laser beam that provides optical feedback for online-stabilization of the axial sample position (*Δz*); the beam traverses the respective objective lens off-axis (solid red path), gets reflected at the embedding medium interface and is imaged onto a camera (dotted red path).

In this study, we devised a coordinate-targeted 4Pi-RESOLFT modality that utilizes negative-switching RSFP (that is, those that are switched off at a wavelength that is also used for generating the fluorescent signal, Fig. 1a) and that resorts to all the processes mentioned above for strong optical sectioning. Concretely, we opted for the RSFP Dronpa-M159T^20^,^21^,^22^, which stands out by relatively fast switching kinetics with comparatively low background. At each scanning position, the local RSFP molecules were cycled through their ON- and OFF-states by consecutive light pulses that defined our RESOLFT imaging sequence (Fig. 1a). In the initial step (‘activation' pulse), we applied a μs-long train of 170–fs pulses at 90 MHz/780 nm in a focal pattern *h*_ac_ to (partially) transfer ('activate') local RSFP to their meta-stable ‘active'-state by two-photon absorption, as described by the activation distribution *S*^B^(**r**). Subsequently, we applied a μs–ms-long ‘deactivation' pulse of continuous-wave (CW) irradiation at 488 or 491 nm, focused to form a hollow deactivation pattern (for example, a ‘z-donut' *h*_zd_, Fig. 1a, Supplementary Methods). This drove active RSFP outside the targeted pixel volume (for example, above and below the focal plane) back to their inactive state, which effectively denied them a further excitation to the fluorescent ON-state and thus improved the spatial ON/OFF-contrast during the following ‘read-out' pulse. We finally probed the remaining active RSFP by a second μs–ms-long CW pulse at 491 nm with a focal pattern *h*_ro_, which transferred them to their fluorescent (ON) state, and detected the fluorescence through a confocal pinhole.

Our scheme thus entails a number of advantages for live-cell 3D-imaging. First, RSFP are inherently live-cell compatible protein markers, and selection of sufficiently bright and stable RSFP is readily available^8^,^20^,^23^. Second, optical sectioning benefits from the additional switching step (activation) involved in the RSFP switching cycle with respect to modalities that do not make use of a meta-stable state. This additional switching becomes especially powerful if it is mediated by two-photon absorption in a 4Pi configuration, as *O*^on^ rises to 4 and the activation and read-out patterns (*h*_ac_, *h*_ro_) can be setup to a limited zone of overlap in the focal region (Fig. 1a). While overlapping several pattern 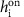 also forms the basis of 4Pi microscopy of type C using two-photon excitation^24^, here we do not require coherent double-lens (4Pi) detection of the emitted fluorescence, and therefore do not need broad-band intra-cavity dispersion compensation. The scheme presented here thus acts to the same effect with much less technical complexity. Finally, activation by two-photon absorption entails much less photo damage than two-photon excitation, as it takes place at a time during the switching cycle when virtually no markers can assume their excited fluorescent state.

**Figure 2:**
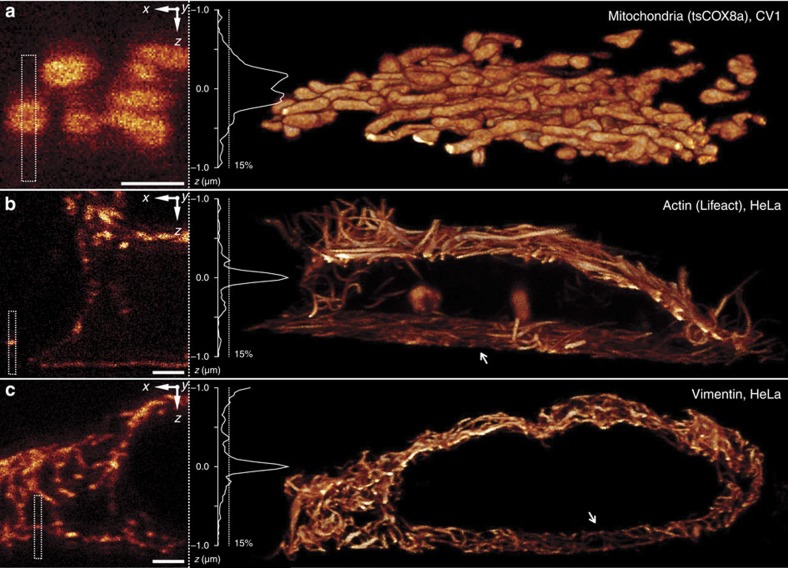
4Pi-RESOLFT imaging exhibits only minor axial lobes. 4Pi-RESOLFT raw data (left) and volume renderings (right) of Dronpa-M159T targeted to (**a**) the lumen of mitochondria, (**b**) actin microfilaments and (**c**) intermediate filaments of the cytoskeleton. The sample in **a** was subject to PFA fixation to freeze the motion of mitochondria; the filament networks in **b**,**c** were recorded from living cells, and exhibit regions of reduced density adjacent to the cover slip (arrows). Estimates of the *z*-response (insets), measured as box profiles over extended structures, exhibit only minor axial lobes in the 15 % range. Fast-to-slow order of scan axes, *xzy*. Pulse parameters, *E*_ac_, *E*_zd_, *E*_ro_=1.6 mW·50 μs, 18 μW·50 μs, 3.1 μW·50 μs. Scale bars, 1 μm.

Under ideal conditions, the effective PSF *h*_ef_ of such a system is virtually free of axial lobes (Fig. 1a) even without a deactivation pulse. In practice, incomplete deactivation and optical aberrations may give rise to lobe amplitudes that are still relevant. To counteract these effects, we applied dedicated lobe deactivation by *h*_zd_ and developed low-aberration^25^, live-cell 4Pi optics (Fig. 1b, Methods). These measures enabled volume scans of over 5-μm-thick mammalian cells without noticeable bleaching at an axial (*z*) resolution in the 100 nm range and axial lobes of only ∼15% of the main peak of the *z*-response, that is, measured on laterally (*xy*) integrated data (Fig. 2). The base acquisition time of 7–21 s μm^−3^ (depending on the brightness of the labelled structure) was short enough to capture the subtly moving cytoskeleton of a living cell (Fig. 2b,c, total acquisition times incl. drift correction overhead b: 115 min per 703 μm^3^, c: 160 min per 400 μm^3^). For highly mobile organelles, such as mitochondria (Fig. 2a), fixation of the sample by paraformaldehyde incubation (Supplementary Methods) offered a means to prevent motion blur. While this treatment irreversibly arrests the cell, its potential to introduce structural artifacts is very low with respect to staining/embedding protocols that involve membrane permeabilization.

**Figure 3:**
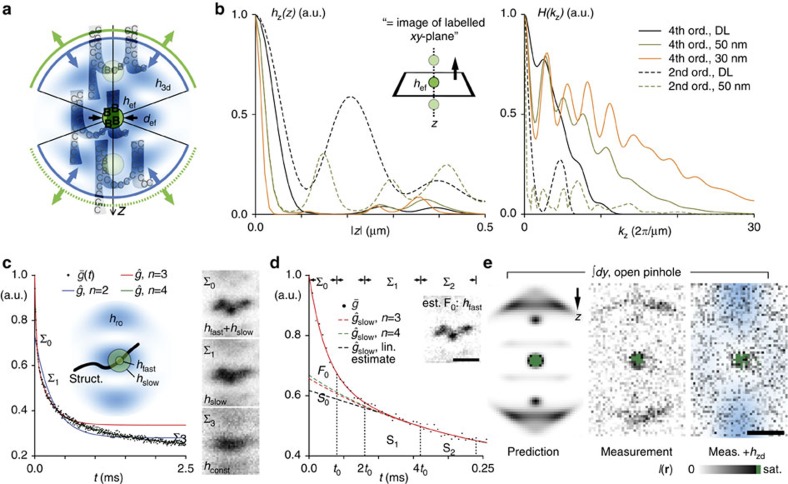
4Pi-RESOLFT image formation with <100 nm isotropic resolution. (**a**) A hollow switching pattern *h*_3d_ confines the central effective PSF to a spot with diameter *d*_ef_ by switching activated markers (B) back to their inactive state (C). Side-lobes due to inefficient switching at low off-centre *h*_3d_ amplitudes rise in relative strength as *d*_ef_ is reduced. *μ*, labelled structure. (**b**) Simulated z-response *h*_z_(*z*) (laterally integrated *h*_ef_) and axial MTF profile *H*(*k*_z_) of the 4Pi-RESOLFT microscope (fourth on-order switching, solid lines) at different target resolutions *d*_ef_. DL, diffraction limit. Graphs for an isoSTED microscope under similar conditions are included for reference (second on-order, dotted lines). (**c**) Normalized time-resolved, mean fluorescence signal 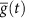 collected from an *xz*-section through an actin fibre bundle (struct.) in a cell expressing Lifeact-Dronpa-M159T. Target resolution 50 nm, read-out pattern *h*_ro_ with a total power of *P*_ro_=3.1 μW incident on the sample. An *n*-component multi-exponential fit to the data corresponds to *n* apparent switching speeds 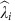. A minimum of *n*=3 is required to adequately represent the data from the beginning of the read-out pulse *t*=0 up to 0.5 ms, 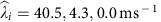 (for up to 2.5 ms: *n*=4, 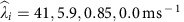). Images Σ_0,1,3_ integrated over time regimes that are dominated by fast (*h*_fast_), slow (*h*_slow_) and about constant PSF components (*h*_const_) exhibit a declining resolution. (**d**) Rate-gated 4Pi-RESOLFT. Extrapolation of the initial contribution of *h*_slow_ (=*S*_0_), based on integrated images Σ_1_ (≈*S*_1_) and Σ_2_ (≈*S*_2_), *t*_0_=40 μs, provides an estimate of the partial image generated by *h*_fast_ (*F*_0_≈ Σ_0_−*S*_0_, inset), improving resolution and image fidelity over Σ_0_. Details are provided in Methods. (**e**) Rate-gated *xz*-sections through actin fibres, recorded with open pinhole to boost out-of-focus signal. The measured (*y*-integrated) side-lobe structure closely resembles the numerical prediction and can be further suppressed (right) by an additional z-donut *h*_zd_ (overlay, *E*_zd_=1.8 μW·1.0 ms). Simulation parameters, numerical aperture 1.20, refractive index 1.362, pinhole diameter 0.5 airy units (**e**: open pinhole). Pulse parameters, *E*_ac_, *E*_3d_, *E*_ro_=1.6 mW·0.2 ms, 1.3 μW·1.6 ms, 3.1 μW·2.5 ms (**e**: 0.5 ms). Scale bars, 250 nm.

To resolve smaller features, we implemented a second switching pattern for deactivation of RSFP around the focal centre: A hollow ‘3D-donut' *h*_3d_ (Fig. 3a), created by a single focused 4Pi beam pair^26^ (Methods), allowed us to tune *h*_ef_ to a near-isotropic resolution below the diffraction limit (Fig. 3a). Calculations using a vectorial diffraction theory^27^ predicted on-axis MTF values of over 40% of the MTF maximum within the MTF bandwidth up to a resolution of 30 nm. This feature keeps the signal well over the noise level in most applications and exemplifies the improvement brought about by higher order on-switching in comparison to modalities of second order such as those reported in isoSTED microscopy^5^ (Fig. 3b). Furthermore, the confinement of the fluorescent on-state, that is, of *h*_ef_, to sub-diffraction 3D volumes means that fewer fluorophores are interrogated at any point in time. This reduction in number of interrogated molecules (that are inherently co-localized) greatly facilitates the quantitative assessment of the properties of the fluorescent labels as they vary in the sample. We found that in time-resolved recordings, the on/off separation contrast decayed over time, hinting to the contribution of multiple deactivation rates. Thus, we introduced a ‘rate-gated' RESOLFT detection scheme that improved both the signal-to-noise ratio in the image and the resolution by discriminating individual signal components (Fig. 3c,d, Methods).

**Figure 4:**
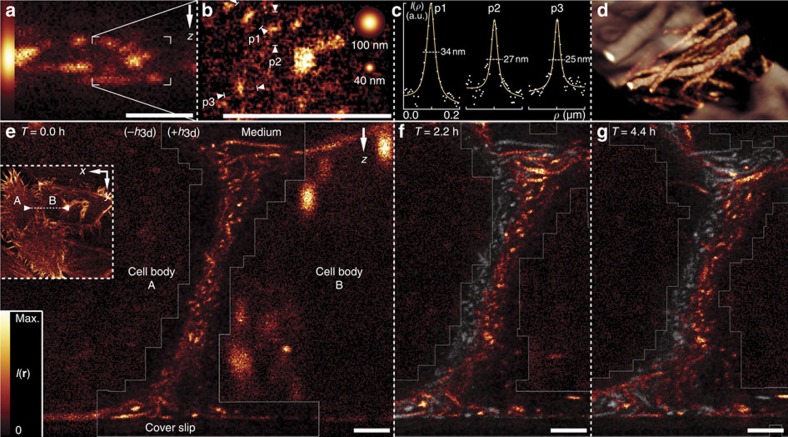
3D nanoscopy with strong optical sectioning. *xz*-sections of live HeLa cells expressing Lifeact-Dronpa-M159T. (**a**) Overview (optical *xz*-section) of actin fibre bundles at an axial base resolution in the 100 nm range. Left inset, confocal reference. (**b**) Addition of a 3D deactivation donut (+*h*_3d_, *E*_3d_=2.6 μW·3.2 ms) to the RESOLFT pulse sequence reveals Dronpa patterns with apparent feature sizes well below 40 nm (inset, Gaussian reference spheres); (**c**) Lorentzian fits, plus a linear local background, to box profiles p1–3 over marked features in **b** along different directions. Numbers indicate full widths at half maximum (FWHM) over background. (**d**) Rendering of the volume surrounding **a**. (**e**–**g**) Time (*T*) evolution of an 8-μm-thick, densely labelled, vertical contact region between two adjacent cells (*xz*-section as marked in the *xy*-overview). Grayscale overlays of the preceding time step (**f**,**g**) aid in the tracking of individual features. A narrowed region of interest was generated online from initial overview scans (−*h*_3d_) at each time frame and imaged at 50 nm target resolution (+*h*_3d_, grey outline, *E*_3d_, *E*_zd_=1.3 μW·1.6 ms, 1.8 μW·0.5 ms). Despite the challenging imaging conditions, stacked actin structures are unambiguously resolved across the full axial extent of the cell layer. *xz*-panels depict rate-gated 4Pi-RESOLFT raw data, solely subjected to noise reduction. Fluorescence intensities *I*(*r*). Common pulse parameters (**b**,**e**–**g**), *E*_ac_, *E*_ro_=1.6 mW·0.2 ms, 3.1 μW·0.5 ms. Scale bars, 1 μm.

Following this approach, we recorded images of Lifeact-Dronpa-M159T-expressing cells and adjusted rate-gating and the RESOLFT pulse sequence for target resolutions of 30-50 nm; the parameters were established by a PSF simulation using measured rate kinetics. Optical *xz*-sections taken perpendicular to the run of solitary actin fibre bundles confirmed the effectiveness of rate-gating (Fig. 3c,d) and the overall shape of the effective PSF (Fig. 3e). Illumination with the z-donut-shaped (*h*_zd_) focus for 1 ms at an average light power of 1.8 μW (488 nm, CW) was sufficient to virtually eliminate lobe background from the image (Fig. 3e, +*h*_zd_), while the low gradients around the central zero of *h*_zd_ with respect to *h*_3d_ facilitated the mutual alignment of these patterns (Supplementary Fig. 1). Turning to the finer structured actin network inside the cell body, we measured apparent feature sizes well below 40 nm (Fig. 4a–c). At a relaxed target resolution of 50 nm and an acquisition time of 3.3 min μm^−2^, we observed the time evolution of the actin scaffold at a vertical contact region of two neighbouring cells (Fig. 4e–g).

## Discussion

Using the current RSFP Dronpa-M159T, rate-gating allowed us to obtain images based on-switching speeds (switch-off half-time *T*^1/2^=10–17 μs at 11.5 kW cm^−2^ illumination intensity, Fig. 3c, Supplementary Fig. 2, Supplementary Table 1) that were over an order of magnitude faster than the previously reported corresponding values for rapid switching RSFP (*T*^1/2^, rsEGFP2: 250 μs (ref. 23), Dronpa-M159T: 230 μs (ref. 28)). Still the recording speed of our 4Pi-RESOLFT nanoscopy scheme can be made substantially faster by parallelization using a multi-spot scanning arrangement. In this case, the recording time of a certain sample area or volume would be cut down by the number of individual recording channels, that is, by the degree of parallelization.

In this study, we opted for cellular structures that are more demanding for 3D-superresolution imaging due to their high spatial density and wide axial extension. Under conditions exacerbated by the optical inhomogeneity of living cells, the signal from a (sub-diffractive) ensemble is easily buried in background (lobe) fluorescence beyond recovery. Nevertheless, owing to the consistently robust MTF of our 4Pi-RESOLFT scheme (Fig. 3b), we obtained raw (Fig. 2, insets) and rate-gated image data (Fig. 4e–g) that were conclusive without the mathematical post-processing (that is, deconvolution) dedicated to lobe-removal that is usually applied in 4Pi-based methods. The actin network seen in the exemplary time-lapse recording (Fig. 4e–g) appeared particularly crowded and extended over 8 μm along the optic axis, which forced the light to pass through several micrometres of cellular material from all angles. Still, the rearrangement of the entwined actin fibres could be traced in great detail, which was possible because the obtained images were practically devoid of axial lobes.

Notably, our scheme of reducing the global refractive index (RI) variance (Fig. 1b) turned out to sufficiently mitigate sample-induced aberrations without adding the complexity associated with adaptive optical elements. The most prominent residual aberration effect was a position-dependent 4Pi phase offset that stemmed from the uneven thickness of the cell layer; it has been accounted for during our recordings by the automated correction mechanism that also counteracted thermal drift (Supplementary Methods).

In conclusion, by realizing 4Pi-RESOLFT nanoscopy based on RSFPs, we have demonstrated exceptional optical sectioning in coordinate-targeted far-field fluorescence nanoscopy, which greatly facilitates nanometre scale 3D fluorescence imaging in living cells. Many accepted constraints to the sample can be lifted, which opens up an imaging regime that has so far been systematically avoided.

## Methods

### 4Pi sample optics for live-cell imaging

In a 4Pi arrangement, the RI (*n*) difference between the material forming a living mammalian cell (*n*≈1.35–1.40) and the surrounding culture medium (typically *n*≈1.33) is a major source of optical aberrations. Aberrations generally reduce the attainable signal-to-noise ratio (*S*/*N*) by blurring the light intensity distributions in the focal region and by raising the intensity of the central minimum of the off-switching light patterns (Figs 1a and 3a). We therefore devised a mounting procedure that minimizes optical aberrations by raising the RI of a standard cell culture medium to *n*=1.362 (Supplementary Fig. 3, Supplementary Methods) and by designing the optical setup accordingly (Fig. 1b, Supplementary Figs 4 and 5): The 4Pi foci are jointly created by two 1.20 NA water immersion objectives that are outfitted with individual tip/tilt-correction to prevent aberrations that arise from lens-coverslip misalignment^29^. The refractive indices of the embedding (*n*=1.362) and immersion media (*n*=1.350), the correction collar settings and the cover slip thickness were chosen to minimize spherical aberrations^25^ over at least 10 μm of sample depth. The changes in the optical path lengths of the two 4Pi-interferometer arms due to *z*-scanning of the sample were compensated^30^ by synchronous position adjustment of the main beam splitter cube (Supplementary Fig. 4).

### A single-focus 3D light pattern for deactivation

To resolve features smaller than 100 nm, we added a RSFP deactivation beam to the microscope. It was imprinted with a circular phase ramp that was subsequently imaged into the back pupil planes of both objective lenses. In contrast to the configuration of a single-lens RESOLFT setup, the direction of rotation of the phase ramp was oriented in countersense with respect to the circular beam polarization at the back pupil planes, which produced a 4Pi off-switching pattern *h*_3d_ that completely surrounded a central zero^26^ (Fig. 3a). Applying *h*_3d_ during the deactivation phase of the RESOLFT switching cycle squeezed the central full width at half maximum (FWHM) *d*_ef_ of *h*_ef_ and allowed us to tune *h*_ef_ to a resolution below the diffraction limit.

### RESOLFT imaging with rate-gated detection

In the present case of RESOLFT imaging of negative-switching RSFP, deactivation of fluorophores during read-out gives rise to a time-dependent signal and hence a time-dependent effective PSF *h*_ef_(**r**,*t*). The time-resolved image g(**r**,*t*) obtained by imaging a structure *s*(**r**) is thus given by


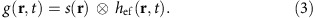


We simplistically assume a deactivation rate *λ*(**r**) that only depends on the read-out intensity *I*_ro_(**r**) and therefore obtain


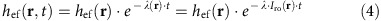


with *t* denoting time relative to the start of the read-out pulse. The deactivation pattern *h*_3d_(**r**) typically confines the effective volume from which fluorescence is collected to a region of FWHM *d*_ef_ around the primary zero of the deactivation pattern. This region is much narrower than the FWHM *d*_ro_ of the diffraction pattern used for read-out:


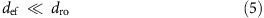


The read-out intensity can then be considered constant, and *h*_ef_(**r**,*t*) follows a mono-exponential decay at a rate that only depends on the peak read-out intensity 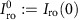. Thus, *g*(**r**,*t*) becomes separable and transforms into:


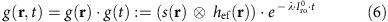


Data from test structures, however, exhibit a distinct multi-exponential behaviour that requires additional components for a proper fit (Fig. 3c):


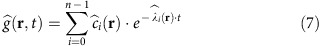


where *n* components with ordered switching rates 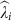, 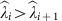 and coefficients 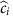 are fitted to the data (‘^' marks fit results). While we attribute the fastest rate 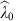 to signal from unimpaired RSFP at the focal centre, the presence of additional rates suggests the co-existence of RSFP species that exhibit significantly slower switching kinetics. A slowed-down switching observed after fixation supports this notion. Unintended processes during image recording also potentially contribute to the observed signal behaviour, for example, the re-activation of RSFP by the read-out light which generates a constant background.

Without loss of generality, we assume a position independent mixture of *n* species with discrete switching rates *λ*_*j*_. Since the deactivation pattern *h*_3d_ shrinks the effective PSF by a *λ*-dependent factor, *h*_ef_(**r**,*t*) has to be generalized to a superposition of *n* individual 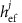 that each correspond to a *λ*_*j*_. Furthermore, we assume our experimental parameters are chosen such that the in-focus part *h*_fast_ of 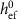 obeys the analogue to equation (5), and pool the remaining contributions in *h*_slow_:


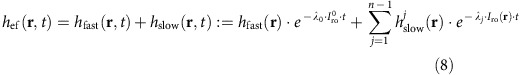


Consequently, the apparent resolution of the acquired image is less than the potential resolution provided by *h*_fast_ and declines over time, as faster components vanish first (Fig. 3c).

To access the full image information that is mediated by *h*_fast_, we implemented an unmixing scheme that isolates the fastest switching signal component (rate *λ*_0_), and that we hence termed ‘rate-gating': according to equation (3), the image generated by a PSF equation (8) takes on the form


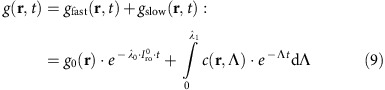


whereby *g*_slow_ is represented by a continuum of exponentials with switching speeds Λ∈[0,*λ*_1_] and coefficients *c*(**r**,Λ) as the result of *s*⊗*h*_slow_.

Hence, a fit 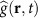 to an imaged structure according to equation (7), in principle, provides a position invariant estimate for 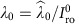 by 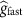, on top of a local approximation of *g*_slow_(**r**,*t*). In practice however, an insufficient photon count often prohibits local fitting of equation (9). We therefore implemented a robust approximation scheme for *g*_fast_(**r**) that only relies on parameters that can be extracted from a fit 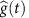 to the global (that is, from a region much larger than the corresponding diffraction limit) spatial average 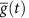 of the measured data:

First, we estimated the time *t*_0_ at which the integrated signal exhibits the maximum *S*/*N* with respect to *g*_fast_:


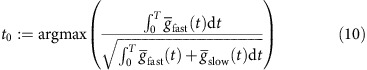


Locally calculated values for *t*_0_ would slightly differ, but as this only affects the statistical error of the result, equation (10) is usually sufficiently precise. Second, we determined a cut-off time *t*_1_ such that


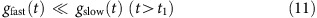


which is usually the case for *t*_1_=2*t*_0_. By further choosing *t*_2_ and *t*_3_ such that *t*_*i*_>*t*_*i*+1_(*i*=0..3), we partition the measured signal into time bins ∑_0,1,2_ (Fig. 3d),


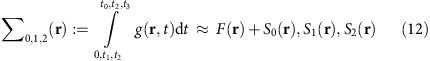


with *F*(**r**) and *S*(**r**) denoting the time integrals over the fast and slow components:


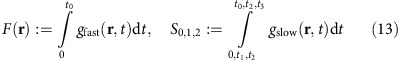


Finally, we estimated *F*(**r**), and thereby *g*_0_(**r**), by linear extrapolation in either zeroth or first order:


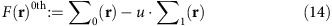



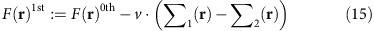


with *u*,*v*=*u*,*v*(*t*_0..3_) denoting geometrical factors that account for the particular choice of the *t*_0..3_. Narrowing the integration intervals defined by *t*_0..3_ and moving them closer to *t*=0, just as the inclusion of the first extrapolation order, reduces the systematic error, but also raises the statistic error due to a reduced photon count. To mitigate this effect, and owing to equation (11), we substituted ∑_1,2_ with their respective resolution-neutral local averages, for example, by applying a Gaussian filter with a FWHM sufficiently far below the FWHM of 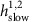.

## Additional information

**How to cite this article:** Böhm, U. *et al.* 4Pi-RESOLFT nanoscopy. *Nat. Commun.* 7:10504 doi: 10.1038/ncomms10504 (2016).

## Supplementary Material

Supplementary Information Supplementary Figures 1-5, Supplementary Table 1, Supplementary Methods and Supplementary References.

Supplementary Movie 1 tsCOX8a-Dronpa-M159T, targeted to mitochondria. Fixed-cell recording, 14.06 x 4.00 x 10.94 μm3 (xzy).

Supplementary Movie 2 Lifeact-Dronpa-M159T, targeted to actin. Live-cell recording, 28.13 x 6.25 x 4.00 μm3 (xzy).

Supplementary Movie 3 Vimentin-Dronpa-M159T. Prior to acquisition, three sample regions (centered at the medial xz-plane of the recorded volume) were deliberately exposed to coincident illumination of the activation and read-out light, which negated the bleaching protection introduced by the 2-photon-activation pulse scheme and consequently resulted in a local signal attenuation. Live-cell recording, 25.0 x 8.0 x 2.0 μm3 (xzy).
