# Supplementary material for: 4Pi-RESOLFT nanoscopy
Source: Nat Commun. 2016 Feb 1;7:10504. doi: 10.1038/ncomms10504 (PMC4740410; doi:10.1038/ncomms10504)
Supplement: Supplementary Information — Supplementary Figures 1-5, Supplementary Table 1, Supplementary Methods and Supplementary References. [file ncomms10504-s1.pdf]

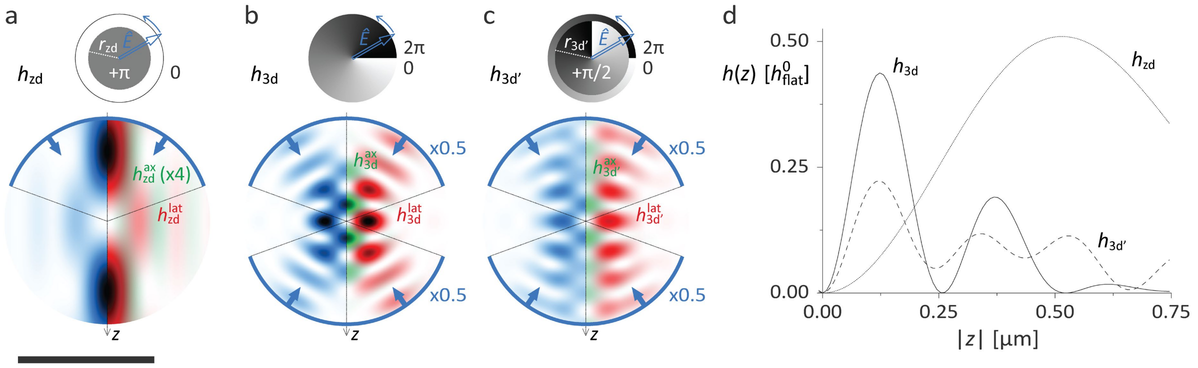

Supplementary Figure 1 | Switching patterns for 3D nanoscopy. Total focal intensity (h, blue) and its decomposition into lateral (h^{lat}, red) and axial (h^{ax}, green) components of three fundamental single-focus switching patterns: (**a**) 'z-donut' (h_{zd}, axial component shown at four times actual strength), (**b**) '3D-donut' (h_{3d}) and (**c**) 'aberrated 3D-donut' ($h_{3d'}$), along their corresponding back pupil phase patterns (top row) and circular polarization states (direction of rotation as marked for an arbitrary field vector $\hat{E}$), normalized to half the peak intensity obtained from single-lens focusing with a flat phase pattern. Phase patterns of opposing lenses (b,c) are mirror images of each other. Radii relative to pupil radius, r_{zd} = 0.71, $r_{3d'}$ = 0.84. (**d**) On-axis profiles. Simulation parameters, numerical aperture 1.20, refractive index 1.362, constant total power, 4Pi phase 0.0. Scale bar, 1 µm.

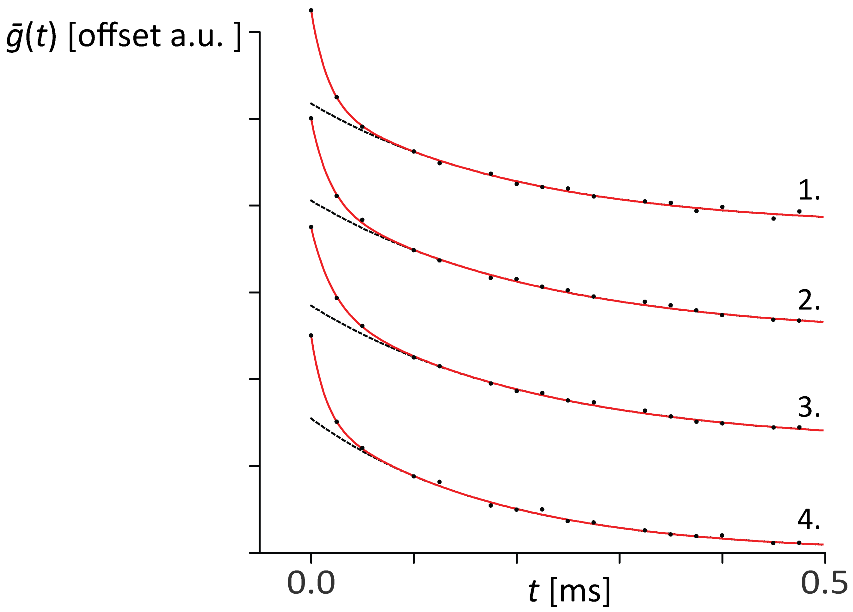

Supplementary Figure 2 | Dronpa-M159T off-switching kinetics in living cells. Multi-exponential fits (red line) to spatial averages $\bar{g}(t)$ of the indicated data sets (1. Fig 3e center, 2. Fig. 3e right, 3. Fig. 4b, 4. Fig. 4e-g), measured at a local read out intensity of 11.5 kW cm^{-2}. Model function $\hat{g}(t) = \sum_{i=0}^{2} a_i \cdot e^{-\lambda_i \cdot t}$, $\lambda_i > \lambda_{i+1}$, $\lambda_2 = 0$. Dashed black lines correspond to the fit less its fastest component (i = 0). Individual parameters are listed in Supplementary Table 1.

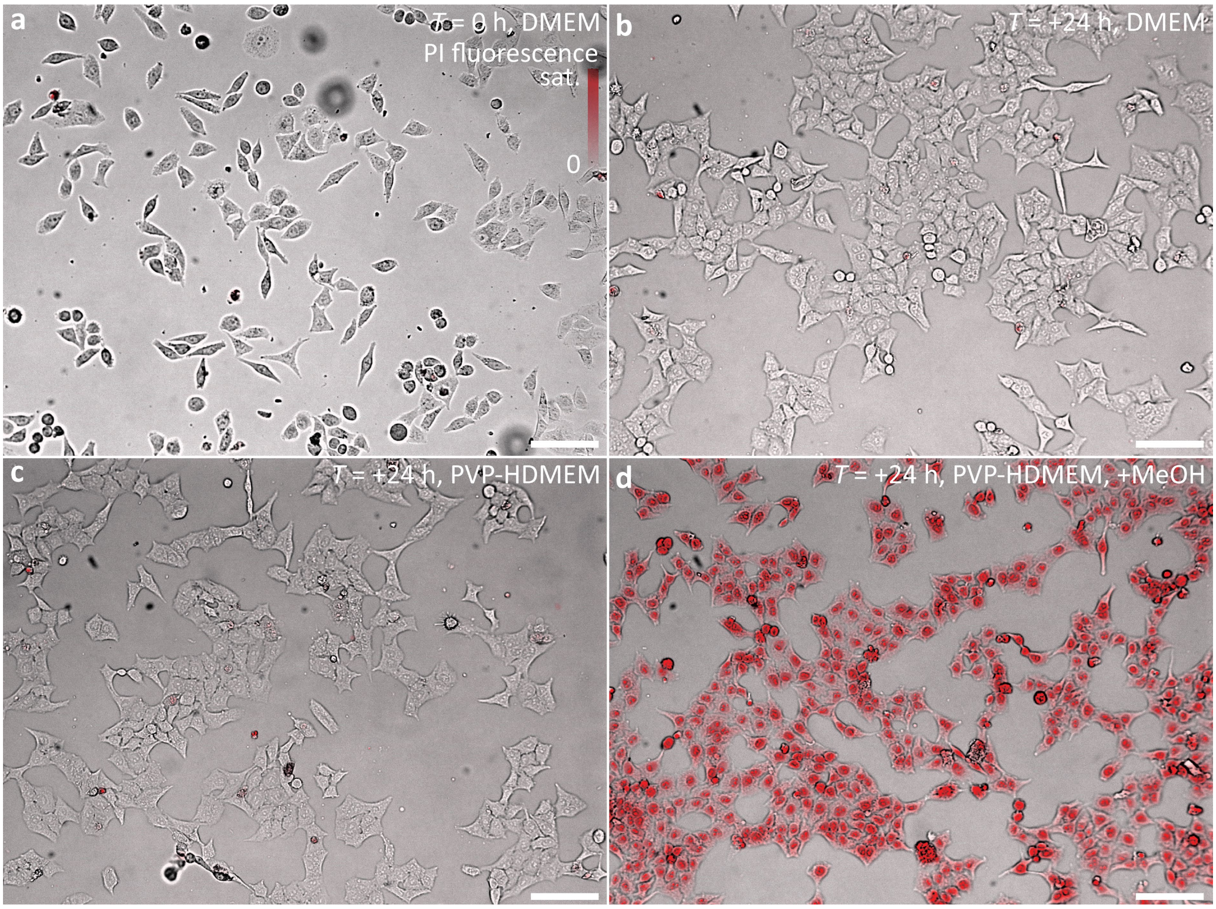

Supplementary Figure 3 | Effect of PVP-HDMEM imaging medium on cell viability. Bright field images with overlaid fluorescence intensities (red) of HeLa cells subjected to a propidium iodide (PI) fluorescence stain, which indicates damaged and dead cells. (**a**) Cultured cells in standard DMEM medium 24 hours after seeding, T = 0 h. (**b**) Cells after further 24 hours of incubation (T = +24 h) in standard DMEM medium. (**c**) Cells at T = +24 h of incubation in PVP-HDMEM imaging medium with refractive index 1.362 exhibit similar viability as cells in (b). (**d**) Positive control. Same coverslip as in (c), recorded after methanol fixation in order to induce cell damage. Scale bars, 100 µm.

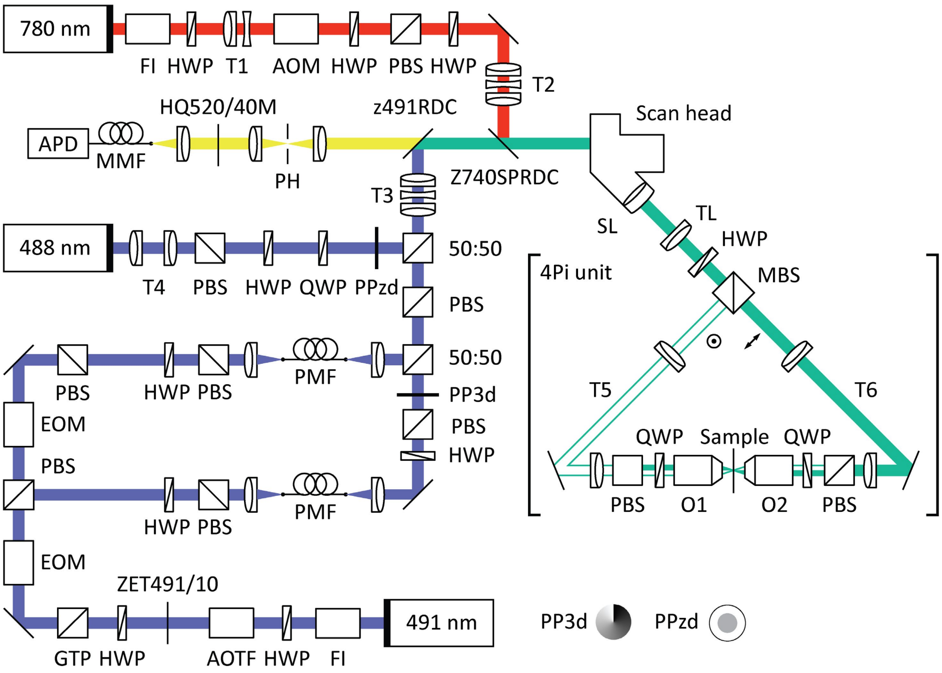

Supplementary Figure 4 | Optical layout of the 4Pi-RESOLFT nanoscope. Acousto-optic modulators AOTF, AOM, electro-optic modulator EOM, Faraday isolator FI, half-wave plate HWP, quarter-wave plate QWP, Glan-Thompson polarizer GTP, polarizing beam splitter PBS, non-polarizing beam splitter 50:50, 4Pi main beam splitter (polarizing) MBS, telescopes T1-6, pinhole PH, polarization-maintaining fiber PMF, multi-mode fiber MMF, scan lens SL, tube lens TL, objective lenses O1, O2, vortex phase plate PP3d, top-hat phase plate PPzd.

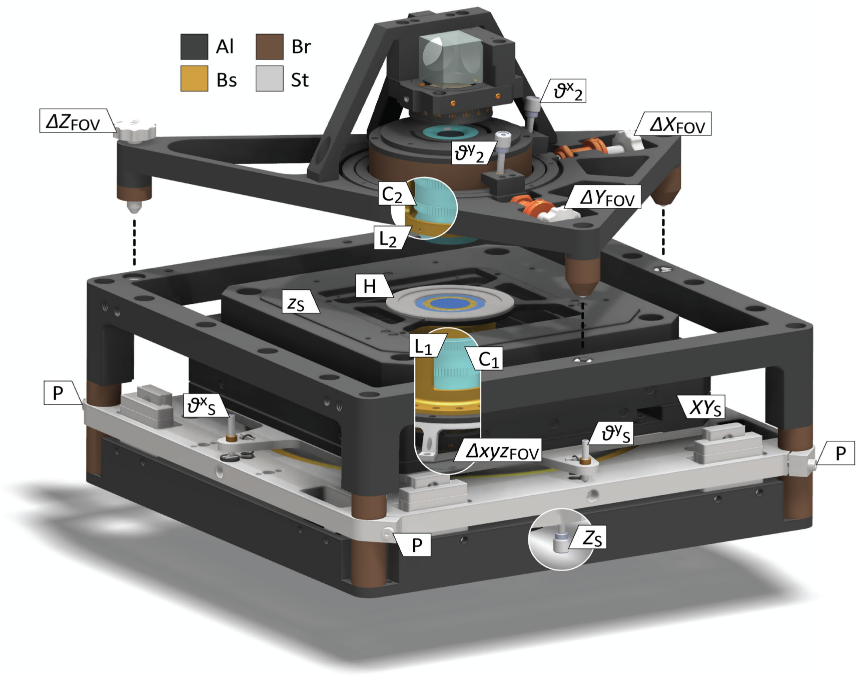

Supplementary Figure 5 | 4Pi unit construction and degrees of freedom. Correction collars $C_{1,2}$, sample holder H, lens cages $L_{1,2}$, clamp screws P, field of view coarse $\Delta X,Y,Z_{FOV}$ and fine Δxyz_{FOV} control, sagittal and meridian sample angles $\vartheta^{x,y}_{S}$ and upper objective angles $\vartheta^{x,y}_{2}$, axial sample position coarse Z_S and fine z_S control, lateral sample position coarse XY_S control. Color coded materials, aluminum alloy Al, bronze Br, brass Bs, stainless steel St.

	Data set	λ_0 [ms^{-1}]	λ_1 [ms^{-1}]	a_0 [1]	a_1 [1]	a_2 [1]	$T_0^{1/2}$ [µs]
1.	Fig. 3e (center)	71	5.6	0.12	0.19	0.69	9.7
2.	Fig. 3e (right)	61	4.9	0.11	0.14	0.75	11
3.	Fig. 4b	56	4.6	0.046	0.076	0.88	12
4.	Fig. 4e-g	47	4.6	0.17	0.29	0.54	15

Supplementary Table 1 | Dronpa-M159T off-switching kinetics in living cells. Parameters of fits depicted in Supplementary Figure 2. Switch-off half-time of fastest component $T_0^{1/2} := \ln(2)/\lambda_0$.

Supplementary Methods

Switching patterns for 3D point scanning nanoscopy

Off-switching patterns used for point scanning fluorescence nanoscopy are characterized by a local intensity minimum at the focal center, flanked by preferably steep gradients. This shape is usually generated by phase modulation of a high-angle focused wavefront, e.g. by means of a phase mask at (or imaged to) the back pupil plane of the objective lens, and/or by control of the phase between the counter propagating wavefront caps that form a 4Pi focus. The following patterns are of particular relevance in context of 3D nanoscopy:

The 'z-donut'[1] (Supplementary Figure 1a) is generated by single-lens focusing of a top-hat shaped wavefront, and was the first switching pattern reported for STED microscopy. By itself, it is a rather inefficient pattern for 3D switching, as most of its intensity is concentrated in two lobes above and below the focal plane, and gradients close to the focal center are comparatively low. These properties, however, make it ideally suited for side-lobe suppression in a two-focus setup (Figs. 1a, 3e).

The '3D-donut'[2] (Supplementary Figure 1b) results from 4Pi focusing of a circularly polarized beam that has been imprinted with an azimuthal phase ramp oriented in countersense to the rotation of the field vector. It represents a very narrow single-focus 3D configuration for ON-state confinement that becomes virtually isotropic at high focusing angles. The precise axial-to-lateral aspect ratio also depends on the distributions of orientation and mobility of the fluorophore dipoles, as the lateral and axial confinement is mediated by orthogonal field components (h^{ax}, h^{lat}, Supplementary Fig. 1b). Secondary minima down to zero intensity are present above and below the focal plane and result in areas of little to no side-lobe suppression.

The 'aberrated 3D-donut' (Supplementary Figure 1c); a 3D-donut that has been modified by a further quarter-wave retardance added to each initial wavefront over a top-hat profile, in order to create a single-focus 3D switching pattern with raised secondary minima and thus improved suppression of on-axis lobes. With respect to a pure 3D-donut, light is diffracted outwards, which flattens gradients by about a factor of 0.5. Just as in case of the z-donut, implementation of the modified phase mask involves a more elaborate alignment due to its dependence on the pupil diameter, which can be facilitated by the use of adaptable phase retarders (e.g. spatial light modulators[3, 4]).

Plasmids

In this study, the term 'Dronpa-M159T' denotes the 'Dronpa-M159T$^{v2.0}$' variant[5] of the reversibly switchable fluorescent protein Dronpa.

Expression plasmids for Lifeact-Dronpa-M159T were generated by cloning of the coding sequences of Lifeact[6] and Dronpa-M159T, connected by the linker GAAGGGGATCCACCGGTCGCCACC, into vector pcDNA3.1(+) (Clontech, Mountain View, CA, USA).

Expression plasmids for Dronpa-M159T-tagged vimentin (Vim) were constructed by Gateway vector conversion (Invitrogen, Carlsbad, CA, USA) from the donor vector pDONR223-Vim.

Expression plasmids for Dronpa-M159T targeted to mitochondria were constructed from the mitochondrial targeting sequence from subunit VIII of human cytochrome c oxidase (from vector pDsRed1-Mito, Clontech).

Cell culture and sample preparation

HeLa (*human epithelial carcinoma*) and CV-1 (*cercopithecus aethiops monkey kidney*) cells were cultured under constant conditions at 37°C and 5 % CO_2 in DMEM (high glucose, Invitrogen, Carlsbad, California) containing 5 % FCS (PAA, Pasching, Austria), 100 µg ml^{-1} streptomycin, 100 U ml^{-1} penicillin, 15 µg ml^{-1} phenol red (all Biochrom, Berlin, Germany) and 1 mM pyruvate (Sigma, St. Louis, USA).

'Large' cover glasses (No. 0, diameter 30 mm, hand-selected thickness 100 µm ±2 µm, Thermo Scientific) were coated with reference beads (FluoSpheres, orange, diameter 100 nm, Life Technologies, Carlsbad, CA, USA) according to a standard Poly-L-lysine protocol[7].

Imaging medium (PVP-HDMEM) stock solution was prepared as a 15.16 % m/m solution of Polyvinylpyrrolidone (PVP40, Sigma-Aldrich, St. Louis, USA) in phenol red-free DMEM culture medium (HDMEM) containing 10mM HEPES (AppliChem GmbH, Darmstadt, Germany). The refractive index was then adjusted to 1.362 and verified by a refractometer. Due to the high molecular weight of the PVP40 (40.000 Da), its contribution to the total concentration of solutes (that governs osmotic pressure) is only on the order of 1 %. In order to assess cell viability in the imaging medium, cells were grown in PVP-HDMEM for 24 hours, subsequently incubated in DMEM with 20 µg ml^{-1} propidium iodide (PI) for 5 minutes, and finally washed with PI-free DMEM. The sample was then examined for PI fluorescence from (dead) cells with membrane defects by fluorescence microscopy (Supplementary Figure 3).

Prior to transfection, cells were seeded onto 'small' coverslips (No. 0, 18 mm x 18 mm, hand-selected thickness 100 µm ±2 µm, Marienfeld, Lauda-Königshofen, Germany) in 6-well plates. On the following day, the cells were transfected with plasmid DNA using Turbofect (Thermo Scientific, Waltham, USA) according to the manufacturer's instructions. 24 hours past transfection, the coverslips were washed for 5 minutes in HDMEM and subsequently covered with 150 µl of a diluted suspension (5 % v/v in HDMEM) of 10 µm beads (FluoSpheres, yellow-green, 10 µm, for blood flow determination, Life Molecular Probes) that stick to the sample and act as spacers. After 5 minutes of incubation, the coverslips were briefly rinsed with HDMEM to remove non-adherent beads, and immersed into imaging medium for another 20 minutes. For paraformaldehyde (PFA) fixation, the respective samples were incubated for 5 minutes in 4 % m/m PFA solution and washed twice in PBS before the immersion medium was applied. 'Small' cover glasses are then stacked on top of their 'large' counterparts, with cells and beads facing inside. The resulting sandwich is gently weighted down in order to bring glass and spacers into contact, sealed by epoxy resin (UHU Plus Sofortfest, UHU, Bühl, Germany), and finally glued into the sample holder (Fig. 1b) using nail polish.

Main optical layout and data acquisition

The microscope was assembled according to Supplementary Figure 4. Light pulses from a Ti:Sapphire laser (Chameleon-XR, Coherent, Santa Clara, CA) were used for two-photon activation of fluorescent

proteins at 780 nm. Light for 3D deactivation (3d) and read-out at 491 nm emanated from a diode-pumped solid state laser (Cobolt Calypso, Cobolt, Vretenvägen, Sweden). A further diode laser (Cobolt MLD, 488nm, Cobolt, Vretenvägen, Sweden) provided a 488 nm beam for the auxiliary deactivation of side-maxima (zd). Individual phase masks were used to imprint phase patterns onto the beams (PP3d: vortex phase plate, VPP-1A, RPC Photonics, Rochester, NY. PPzd: top-hat phase plate, MgF_2 deposited onto fused silica, custom built in our optics workshop), that were subsequently imaged to the back pupil (BP) of each objective lens (1.20 NA HCX PL APO, 63x, Leica Microsystems, Wetzlar, Germany) in order to generate the desired focal light patterns (Supplementary Figure 1). Scanning of the sample was implemented by a galvanometric beam scanner (Yanus IV, TILL Photonics, Gräfeling, Germany), located at a conjugated plane with respect to both BP planes, and a piezo driven sample stage (P-541.ZCD, Physik Instrumente, Karlsruhe, Germany) with added optical stabilization (Fig. 1b).

Data acquisition was controlled by a Python-scripted Imspector framework (Imspector, MPIbpc, Göttingen, Germany) running on a personal computer. Physical connection to downstream hardware (e.g. trigger logic, scanner, 4Pi slow control) and the detector (avalanche photo diode, SPCM-AQR-13-FC, PerkinElmer, Salem, MA) was provided by two I/O-modules (PCI-6259, National Instruments, Austin, Texas, USA) and a USB interface. Pulse sequences for RESOLFT imaging were shaped by acousto- and electro-optical light modulators connected to a pulse generator (Model 9518, Quantum Composers, Bozeman, MT, USA).

4Pi unit operation

The construction of the 4Pi unit was geared to combine convenient handling with minimal drift during measurements. Its geometry and material composition (Supplementary Figure 5) were chosen such that the thermal expansion of corresponding components cancels out at the focus position in order to minimize thermal drift. The rigidity of each objective lens was increased by a lens cage ($L_{1,2}$) that fixes the movable lens cap to its base.

Optical contact between each objective lens and the sample was established by a droplet of refractive index liquid (Series AAA, n_D = 1.350, Cargille Laboratories, Cedar Grove, NJ, USA). The individual PSFs of each objective lens as well as their joint 4Pi PSF were deduced from images of fluorescent reference beads inside the sample. The initial alignment of the unit (Supplementary Figure 5) adhered to standard procedures[8] and involves minimizing the PSF extends by means of the correction collars ($C_{1,2}$), straightening of the PSFs by collinear alignment of the objective lens and sample axes (ϑ_2, ϑ_S), and coarse superposition of the fields of view (FOV) of both lenses ($\Delta X,Y,Z_{FOV}$).

During routine operation, the upper objective mount was detached in order to change the sample, and subsequently reattached with about 2 µm accuracy, which lies well within the travel of the lower objective piezo stage (30 x 30 x 10 µm, P-733.3DD, Physik Instrumente, Karlsruhe, Germany) used for FOV fine control (Δxyz_{FOV}). Sample orientation (ϑ_S) and coarse axial position (Z_S) were realigned as necessary. Axial travel of the goniometer platform could be locked against the outer frame by four clamp screws (L) for improved long term stability. An automated online (drift) correction for the 4Pi phase ($\Phi_{4\mathrm{Pi}}$) and diverging FOV was implemented as a Python script within the data acquisition framework. Scanning was periodically switched from the region of interest (ROI) to the location of predefined, 100 nm reference beads, in order to assess the current $\Phi_{4\mathrm{Pi}}$ and FOV mismatch. The

corresponding corrections were then applied to the main 4Pi beam splitter (MBS, Supplementary Figure 4) and $\Delta xyz_{\mathrm{FOV}}$, and scanning of the ROI finally resumed.

A notable feature of the optical layout of the 4Pi unit (Supplementary Figure 4) is the suppression of the transmitted light. In a typical 4Pi arrangement, light that is focused on the sample is usually picked up and re-collimated by the opposing objective lens, and therefore directed towards the detector where it needs to be filtered out in order to prevent it from generating image background. Here, due to the polarization state of the transmitted light, it is already attenuated by a factor of 10^{-1}-10^{-2} at the polarizing beam splitter adjacent to the respective objective lens (Supplementary Figure 4, 4Pi unit, hollow and solid beam paths), which relaxes the requirements on further filters.

Supplementary References

1. Klar TA, Jakobs S, Dyba M, Egner A, Hell SW. Fluorescence microscopy with diffraction resolution barrier broken by stimulated emission. *Proc Natl Acad Sci USA* **97**, 8206-8210 (2000).

2. Schmidt R. 3D fluorescence microscopy with isotropic resolution on the nanoscale. PhD thesis, University of Heidelberg (2008).

3. Willig KI, Rizzoli SO, Westphal V, Jahn R, Hell SW. STED microscopy reveals that synaptotagmin remains clustered after synaptic vesicle exocytosis. *Nature* **440**, 935-939 (2006).

4. Auksorius E*, et al.* Stimulated emission depletion microscopy with a supercontinuum source and fluorescence lifetime imaging. *Opt Lett* **33**, 113-115 (2008).

5. Willig KI, Stiel AC, Brakemann T, Jakobs S, Hell SW. Dual-Label STED Nanoscopy of Living Cells Using Photochromism. *Nano Lett* **11**, 3970 - 3973 (2011).

6. Riedl J*, et al.* Lifeact: a versatile marker to visualize F-actin. *Nat Methods* **5**, 605-607 (2008).

7. Wurm CA, Neumann D, Schmidt R, Egner A, Jakobs S. Sample preparation for STED microscopy. *Methods Mol Biol* **591**, 185-199 (2010).

8. Schmidt R, Engelhardt J, Lang M. 4Pi Microscopy. *Methods Mol Biol* **950**, 27-41 (2013).
